# Eosinophils Respond to Extracellular Matrix Treated Muscle Injuries but are Not Required for Macrophage Polarization

**DOI:** 10.1002/adhm.202400134

**Published:** 2024-07-27

**Authors:** Ravi Lokwani, Daphna Fertil, Devon R. Hartigan, Aditya Josyula, Tran B. Ngo, Kaitlyn Sadtler

**Affiliations:** ^1^ Section on Immunoengineering Center for Biomedical Engineering and Technology Acceleration National Institute of Biomedical Imaging and Bioengineering National Institutes of Health Bethesda MD 20892 USA

**Keywords:** eosinophils, immunoengineering, muscle trauma, regeneration, wound healing

## Abstract

The immune response to decellularized extracellular matrix (ECM) muscle injury is characterized by Th2 T cells, Tregs, M2‐like macrophages, and an abundance of eosinophils. Eosinophils have previously been described as mediators of muscle regeneration but inhibit skin wound healing. In addition to response to wounding, a large number of eosinophils respond to biomaterial‐treated muscle injury, specifically in response to decellularized ECM. ECM treatment of muscle wounds has been associated with positive outcomes in tissue regeneration, but the detailed mechanisms of action are still being evaluated. Here, this work investigates the role of these eosinophils in terms of their immunologic phenotype and subsequent effect on the local tissue microenvironment. These cells have a mixed phenotype showing both type‐2 and regulatory gene upregulation and but are not required for macrophage polarization. Beyond the local tissue, ECM treatment is seen to induce a transient flux of eosinophils to the lungs but prevented a trauma‐associated neutrophilia in the lungs of injured mice. This work believes this local and systemic immunomodulation contributes to the regenerative effects of the material and such distal tissue effects should be considered in therapeutic design and implementation.

## Graphical abstract

Eosinophils rapidly respond in very high abundance (≈30–50% of all immune cells) to decellularized extracellular matrix (ECM) treated muscle injuries. In this study, their role is evaluated in shaping macrophage polarization and tissue architecture in the early stages of immune response to injury when these cells are the most prevalent.

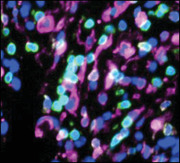

## 1 Introduction

There is a consensus in bioengineering that the outcome of a device used to repair or replace the function of a damaged tissue or organ is strongly dependent upon the immune system.^[^ ^1^ ^]^ Immune cells can have both pro‐healing and rejection phenotypes when it comes to biomaterials and recent efforts have focused on controlling and manipulating these responses to promote tissue integration and regeneration. When responding to an insult such as a wound or foreign body, the first cells to respond to the injury are granulocytes, such as neutrophils and eosinophils, and macrophages.^[^ ^2^ ^]^ There is a robust body of literature on the role of macrophages in wound healing and the foreign body response, but less is known for granulocytes especially with biomaterial‐mediated tissue regeneration. Eosinophils have been implicated on recovery from denervation‐based (cardiotoxin‐induced) muscle injury, and neutrophils are known responders in a canonical foreign body response (FBR).^[^ ^3^ ^]^

Initial neutrophil dominated inflammation is a part of typical pro‐fibrotic FBR that precedes collagen encapsulation of implanted material.^[^ ^4^ ^]^ Whereas we had previously reported a robust neutrophil recruitment to certain materials such as polyethylene and polyethylene glycol, the decellularized extracellular matrix (ECM) biomaterial has a robust eosinophil recruitment.^[^ ^5^ ^]^ The type‐2 like response against biologically‐derived scaffolds such as decellularized ECM are documented as pro‐regenerative in nature due to its tissue regeneration associated inflammatory components such as presence of pro‐regenerative macrophages and adaptive IL‐4 secreting Th‐2 cells that promote macrophage class switching into pro‐regenerative phenotype.^[^ ^6^ ^]^ Besides macrophages, tolerogenic dendritic cells, basophils, and adaptive immune cells, the biological scaffold induces a very high influx of eosinophils at the site of implant that makes up a significant fraction of the immune cells responding to the material.^[^ ^5^, ^7^ ^]^ As these signals have also been associated with fibrosis, a delicate balance must be struck in designing new materials to promote proper tissue regeneration and work in concert with other immune cells such as regulatory T cells that have been directly implicated in this regenerative process.^[^ ^8^ ^]^

While a significant amount is known about the pro‐inflammatory role of eosinophils in disease such as asthma,^[^ ^9^ ^]^ esophagitis,^[^ ^10^ ^]^ and helminth infection,^[^ ^11^ ^]^ not much is known about their role in biomaterial tissue interaction especially in relation to their abundant presence at biological scaffold implant site. Under healthy state, bone marrow generates low number of eosinophils into circulation that home to gut mucosa where they help in maintenance of homeostasis.^[^ ^12^ ^]^ Recent findings have suggested that eosinophils residing in tissue under healthy state express an immuno‐regulatory profile compared to inflammatory eosinophils released in circulation due to allergic reaction such as type‐2 asthma.^[^ ^13^ ^]^ Past studies have characterized inflammatory and regulatory eosinophils based on surface expression of CD101 (a costimulatory marker that has also been associated with regulation of colitogenic T cells^[^ ^14^ ^]^) and CD62L (L‐selectin‐ adhesion molecule) expression where inflammatory eosinophils phenotype had CD101^high^ and CD62L^low^ expression and were featuring segmented nucleus morphology whereas regulatory eosinophils had ring‐shaped nucleus along with CD101^low^ and CD62L^moderate^ surface expression.^[^ ^13^ ^]^ Given the ring‐shaped morphology of most eosinophils in mice (compared to humans) it is still possible to evaluate these phenotypes in mice through the expression of CD101 and CD62L.

Understanding more about the mechanisms of action of these “pro‐regenerative” materials could lead to optimized formulations that promote a more robust tissue regeneration.^[^ ^5^, ^6^ ^]^ It is likely that there are components of responses to ECM scaffolds that are both pro‐regenerative but also components that could be reduced or augmented to increase new tissue growth and decrease scar tissue formation. Given the dual role of type‐2 immune responses (such as those associated with eosinophilic infiltration) in promoting would healing but also being associated with fibrosis,^[^ ^15^ ^]^ we sought to characterize the granulocyte response to decellularized ECM. This allows us to determine if the robust eosinophil infiltration was more canonically inflammatory or serving a more nuanced role in the wound healing and regeneration process. As others have reported a critical role for eosinophils in muscle healing in a fundamentally different muscle injury model—cardiotoxin injection—we sought to understand the potential role of ECM‐induced eosinophil recruitment in its pro‐regenerative phenotype. Our goal in this study was to understand the kinetics of recruitment, immunological function of muscle eosinophils, and relevance to muscle regeneration in the context of traumatic muscle injury and material implantation for reconstructive purposes.

## 2 Results and Discussion

**Figure 1:**
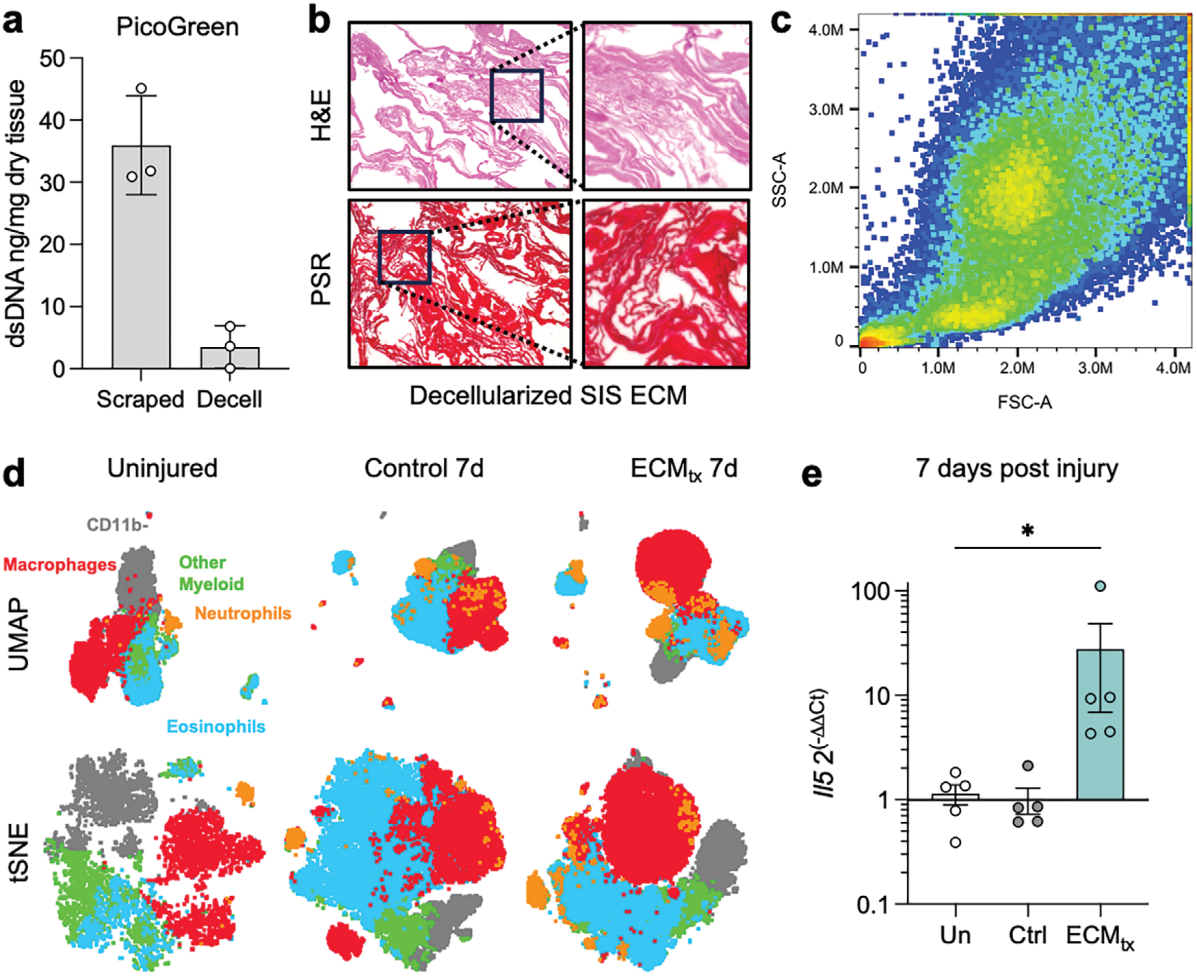
Decellularization of extracellular matrix (ECM) and overall infiltrate into muscle injury. a) PicoGreen double‐stranded DNA (dsDNA) quantification in mechanically scraped small intestinal submucosa (SIS) and decellularized SIS (Decell) as a fraction of lyophilized dry weight. Data are mean ± standard deviation, *n* = 3 different lots of ECM. b) Hematoxylin and eosin (H&E, top) and picrosirius red (PSR, bottom) stained decellularized SIS ECM. c) Cell infiltrate into muscle at 7 days post‐injury (dpi) by flow cytometry. FSC = forward scatter, SSC = side scatter. Representative image of *n* = 5. d) Overall immune infiltrate at 7 dpi detected by flow cytometry and displayed by dimensionality reduction algorithms, UMAP (uniform manifold approximation and project) and tSNE (t‐stochastic neighbor embedding). Gray = CD45+CD11b‐ cells, Green = CD45+CD11b+Lin‐ myeloid cells, Red = F4/80+ macrophages, Orange = Ly6G+ neutrophils, Blue = SiglecF+ eosinophils. e) Gene expression of Il5 (eosinophil activation cytokine) in muscle quantified as a fold change over uninjured control. Data are mean ± SEM, *n* = 5 per treatment group, ^*^ *p* < 0.05, Kruskall–Wallis ANOVA with Dunn post‐hoc correction for multiple comparisons.

As previous studies revealed a robust eosinophil response to decellularized ECM,^[^ ^5b^ ^]^ we sought to characterize this response through a murine model of volumetric muscle loss. Given clinical uses of porcine decellularized small intestinal submucosa (SIS) in the clinic and minimal difference between skeletal muscle derived and SIS derived matrices,^[^ ^16^ ^]^ we sought to evaluate the clinically used ECM product (SIS) in our studies. We prepared ECM through mechanical isolation of the SIS followed by treatment with peracetic acid that yielded a significant decrease in double stranded DNA as quantified by PicoGreen assay (*p* = 0.0029, **Figure** **1**a). Decellularization was subsequently confirmed through histologic analysis of the ECM revealing the absence of hematoxylin‐stained nuclei via hematoxylin and eosin (H&E) and staining of collagen with picrosirius red (PSR, Figure 1b). After VML and treatment with ECM for 7 or 21 days, we excised the quadriceps muscle and generated a single cell suspension followed by staining with a 10‐color flow cytometry panel to evaluate immune cell infiltration, activation, and polarization—focusing on granulocytes (Figure 1c,d). Through flow cytometry there was an obvious and abundant population of high side‐scatter cells that is characteristic of granulocytes such as neutrophils and eosinophils (Figure 1c). This was confirmed with staining for Siglec‐F where we saw a large presence of eosinophils (Figure 1d), accompanied by a significant increase in *Il5* gene expression upon treatment of the injury with ECM (ECM_tx_, Figure 1e). IL‐5 is a cytokine important in the activation of eosinophils that is secreted by both immune and nonimmune cells. While IL‐5 is associated with a conversion of M1‐like macrophages to M2‐like macrophages that aid in wound healing, healing of skin wounds in IL‐5 overexpressing mice was shown to be inhibited and characterized by an excessive infiltration of eosinophils.^[^ ^17^ ^]^ There is less known directly on the role of IL‐5 in muscle wound healing as opposed to skin healing where it has been targeted via monoclonal antibody blocking to promote wound repair.^[^ ^18^ ^]^ IL‐5 is associated with some pro‐fibrotic type‐2 immunity, but likely plays multiple roles that require precise timing for optimal outcomes.

### 2.1 Eosinophil and Macrophage‐Dominant Inflammation in Response to ECM‐Treated Muscle Injury

**Figure 2:**
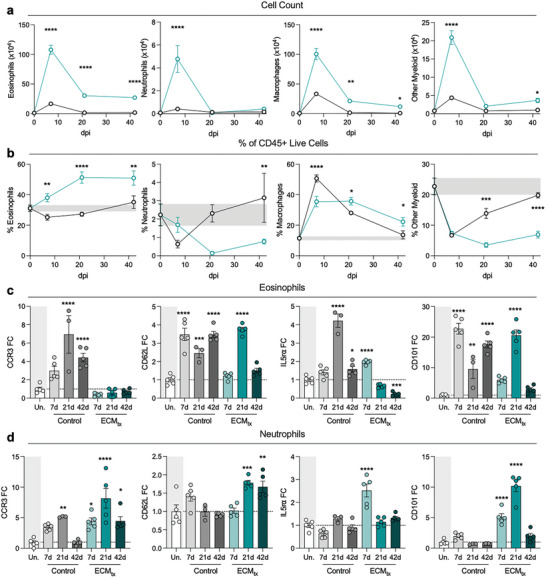
Granulocytic response to injury with and without extracellular matrix (ECM) treatment. a) Count of immune cells in response to injury per quad, t0 = uninjured. b) Proportion of myeloid cells in response to injury as a fraction of CD45+ cells. Teal = ECM_tx_, Black = control injury, grey band = uninjured range. c,d) Fold change of CCR3, CD62L, IL5ra, and CD101 fluorescence intensity over cells isolated from uninjured mice at 7‐, 21‐, and 42‐days post‐injury on c) eosinophils, and d) neutrophils. White bar = uninjured, Black/gray bars = control injury, Teal bars = ECM_tx_. Data are mean ± SEM, *n* = 3–5 mice. ANOVA with FDR or Tukey post‐hoc correction for multiple comparisons. ^*^ *p* < 0.05; ^**^ *p* < 0.01; ^***^ *p*< 0.001; ^****^ *p* < 0.0001 compared to uninjured control.

Consistent with past findings on presence of eosinophils and neutrophils in different tissues during steady‐state,^[^ ^13^, ^19^ ^]^ we reported a small presence of eosinophils, macrophages along with few neutrophils and other immune cells in an uninjured mouse quadriceps muscle (*t* = 0, **Figure** **2**a,b). As expected, there was a large increase in overall immune infiltration after VML that persisted through 42 days post‐injury which was enhanced by material treatment and dominated by large numbers of myeloid cells (CD11b^+^) including eosinophils (CD45^+^CD11b^+^Siglec‐F^+^F4/80^lo^Ly6G^−^SSC^hi^) and macrophages (CD45^+^CD11b^+^Siglec‐F^−^F4/80^+^Ly6G^−^SSC^hi^, Figure 2a). There was also a smaller but significant infiltration of neutrophils (CD45^+^CD11b^+^Siglec‐F^−^F4/80^−^Ly6G^+^SSC^hi^) in both control and ECM_tx_ injuries that rapidly peaked at 7 days post‐injury (dpi) then returned to baseline within 21 dpi. The persistence of this proportion of eosinophils is one of the most robust phenotypes of this material alongside the M2‐like macrophages that accompany it, which have been previously described as expressing a number of scavenger receptors including CD206 and CD301b.^[^ ^5^, ^6^ ^]^

While numbers of immune cells increase after injury, as a fraction of total immune cells (CD45^+^) the eosinophil proportion saw a decline from its uninjured level in response to control injury at 7 dpi and continued until 21 dpi before rising again to uninjured levels at 42 dpi (Figure 2b). In contrast to control injury, the ECM‐treated muscle injury displayed a significant (*p* < 0.01) increase in eosinophil recruitment which peaked at 21 dpi ($\bar{X}=51.28\%\pm \mathrm{SEM}=3.77$) and was maintained through 42 dpi. Both control and ECM_tx_ injuries bore strong persistence of macrophages throughout injury recovery with the control injuries having slightly higher macrophage prevalence at 7 dpi which correlated with the higher eosinophil response to ECM_tx_. While control injury returned close to steady‐state uninjured immune infiltrate by 42 dpi (both in cell count and proportions of CD45+ cells) ECM_tx_ injury was still undergoing immune responses through 42 dpi in agreement with prior studies.^[^ ^5b^ ^]^ This large increase in eosinophil infiltration to an injury site is as high as those seen in standard eosinophil responses such as asthma and allergy.^[^ ^20^ ^]^ Given their strong ability to alter local microenvironments through secretion of cytokines and chemokines as well as degranulation with lipases, collagenase, cathepsin, and ribonucleases, this rapid and large influx into the wound space likely alters the other immune cells in that space as well as the resident stem cells.

### 2.2 Alterations in Activation Markers and Cytokine/Chemokine Receptors on Granulocytes After Injury and ECM Treatment

Previous studies have reported different phenotypes of granulocytes based on surface expression of CD62L, CD101, CCR3, and IL5ra in steady state and in inflammatory conditions.^[^ ^13^ ^]^ We decided to utilize these markers to understand the change in granulocyte phenotypes from their homeostatic state (uninjured muscle) in response to injury with and without ECM treatment during the course of initial injury response and the resolution timeframe (Figure 2c,d). The eosinophils recruited in response to control injury expressed higher levels of CCR3 (that can bind both eotaxin and eotaxin‐3 along with MCP3, MCP‐4 and RANTES) in comparison to those isolated from an uninjured muscle. This expression level peaked at 21 dpi but remained elevated at 42 dpi. In contrast, CCR3 expression did not significantly increase on eosinophils in ECM_tx_ injuries and remained constant throughout the course of injury recovery. Eosinophils in response to both control injuries and ECM_tx_ injuries saw increases in their CD62L expression with a more transient upregulation seen in ECM_tx_ that peaked at 21 dpi. High CD62L expression has been associated with more anti‐inflammatory resident eosinophils in the lung whereas CD62L low cells are associated with inflammatory peripheral blood recruited eosinophils.^[^ ^21^ ^]^ As with CCR3, IL5ra saw greater increases on eosinophils in response to control injuries peaking at 21 dpi, whereas ECM_tx_ saw an early peak at 7dpi with a refractory decrease by 42dpi. IL‐5, the ligand for IL‐5ra, is a pro‐inflammatory cytokine that has been associated with the pathology of eosinophilic diseases.^[^ ^22^ ^]^ Expression of CD101 mirrored the patterns of CD62L expression with a persistent upregulation in control injuries, whereas ECM_tx_ saw a transient peak at 21 dpi. As mentioned, CD101 has previously been reported to act to limit the responses of colitogenic T cells, possibly acting in a regulatory role which may also be occurring in response to wounding.^[^ ^14^ ^]^

The pattern of expression of these receptors differed on neutrophils (Figure 2d). Both control and ECM_tx_ injuries had peaks of CCR3 expression by 21 dpi but ECM_tx_ maintained higher CCR3 expression both at 7 and 42 dpi. Expression of CD62L on neutrophils did not increase significantly in control injury but was higher in ECM_tx_ neutrophils at both 21 and 42 dpi, albeit with lower fold changes over uninjured controls in comparison to the changes seen on eosinophils. While eosinophils in control injury saw an increase in IL5ra, the same was not true for neutrophils; however, we did see similar IL‐5ra increases at 7 dpi in ECM_tx_ neutrophils. The same was true for CD101 expression, with minimal changes from uninjured in the control injuries, but significant increases in CD101 expression on neutrophils at both 7 and 21 dpi in ECM_tx_ injuries. The expression of both CD62L and CD101 on neutrophils that are in the lungs of ECM_tx_ mice is of interest as both of these makers have been associated with a less inflammatory phenotype than their CD62L‐CD101‐ counterparts.

### 2.3 Type2/Regulatory Transcriptome is Induced in Eosinophils Responding to ECM_tx_ Injury

**Figure 3:**
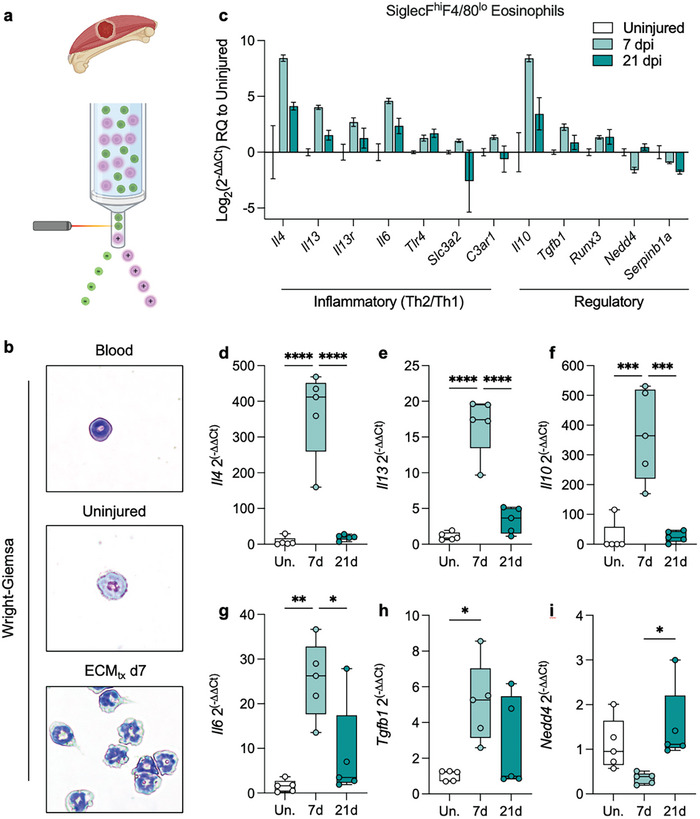
Transcriptome of injury responding eosinophils has a type‐2 and regulatory profile. a) Schematic of cells isolated for analysis, made in BioRender. b) Phenotype of eosinophils isolated from peripheral blood, uninjured mouse quad, and injury with extracellular matrix (ECM) treatment after 7 days. c) Gene expression in eosinophils isolated from ECM_tx_ muscle injuries compared to eosinophils from uninjured mice at 7‐ and 21‐days post injury. Data are mean ± SEM. d–i) Significantly altered genes in eosinophils from ECM_tx_ mice d) *Il4* encoding Interleukin (IL) 4, e) *Il13*, f) *Il10*, g) *Il6*, h) *Tgfb1*, i) *Nedd4*. Box plots are min to max and interquartile range, *n* = 5 mice. ANOVA with Tukey post‐hoc correction for multiple comparisons. ^*^ *p* < 0.05; ^**^ *p* < 0.01; ^***^ *p* < 0.001; ^****^ *p* < 0.0001 compared to uninjured control.

Given the strong prevalence in response to ECM_tx_ injury and in order to further characterize the eosinophil response to these injuries we sorted them from uninjured muscles and ECM_tx_ injuries at 7 and 21 dpi. The eosinophil sorted from blood, uninjured muscle, or from ECM‐treated injured muscle had a consistent ring‐shaped nucleus morphology characteristic of murine eosinophils (**Figure** **3**b). The eosinophils sorted from ECM_tx_ muscle injury did display a more activated (hypersegmented nuclei) and degranulated phenotype (minimal eosin staining in cytoplasm) in comparison to those from uninjured muscles and the peripheral blood. The eosinophils in ECM‐treated injury at 7 dpi had significantly elevated expression of *Il4*, *Il13*, *Il10* and *Il6* in comparison with both uninjured muscle and ECM‐treated injury at 21 dpi (Figure 3d–g) and *Tgfb1* in comparison with uninjured only (Figure 3h). The eosinophils from ECM‐treated injury at 21 dpi had significantly elevated *Nedd4* (previously associated with tolerance and immunoregulation^[^ ^23^ ^]^) expression in comparison with ECM‐treated injury at 7 dpi (Figure 3i). This upregulation of *Il6*, *Il4*, *Il13*, and *Il10* in response to ECM scaffolds has previously been described in T cells within the muscle wound microenvironment.^[^ ^5b^ ^]^ Induction of IL‐4 has been reported systemically as well with upregulation in draining lymph nodes.^[^ ^6a^ ^]^

The products of these genes have been implicated in multiple stages of tissue regeneration and wound healing including IL6 interacting with satellite cells to promote muscle hypertrophy,^[^ ^24^ ^]^ IL‐4 mediated myoblast fusion to form multinucleate myofibers,^[^ ^25^ ^]^ IL‐13 effecting metabolism during exercise,^[^ ^24b^ ^]^ and IL‐10 associated with Treg function which has been shown to regulate macrophage behavior and regenerative outcome in a cardiotoxin model.^[^ ^8^, ^26^ ^]^ There was also an upregulation of the gene encoding TGFβ, which has been implicated both in regulatory immune responses and in fibrosis.^[^ ^27^ ^]^ The expression of a heterogeneous set of immune factors could point at a unique polarization state, or a multitude of eosinophil subsets that is suggested by the differential surface markers shown in Figure 2. While these markers have shown both the potential for pro‐regenerative and pro‐fibrotic phenotypes, it is likely that these cells play a role in the regeneration process. Previous reports have shown that IL‐4 derived from eosinophils was required for regeneration after cardiotoxin (snake venom) mediated muscle degeneration, suggesting a potential conserved role of eosinophils in production of IL‐4 in response to both chemically induced and physically induced muscle damage.

### 2.4 Systemic Effects of Local Material Treatment in Muscle Injury are Observed in the Peripheral Blood and Lung

**Figure 4:**
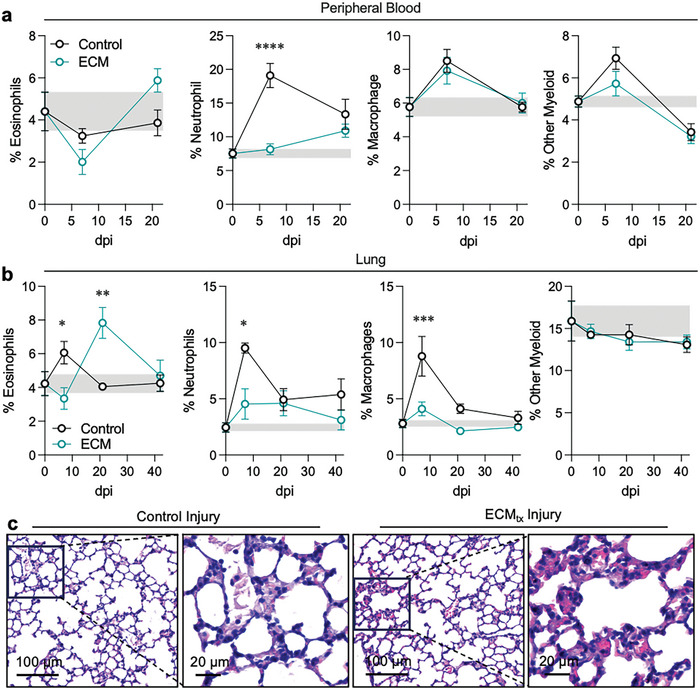
Systemic alterations in granulocyte response extend to lung tissue from distal muscle injury. a) Proportion of CD45+ cells that are eosinophils (SiglecF+), neutrophils (Ly6G+), Macrophages (F4/80+), and other myeloid cells (CD45+CD11b+Lin‐) in a) peripheral blood and b) lung tissue of mice. Teal = ECM_tx_, Black = control injury, grey band = uninjured range. dpi = days post injury. c) Hematoxylin and eosin (H&E) stained sections of mouse lung at 7 days post‐injury (dpi) in wild‐type (WT) mice. Data are mean ± SEM, *n* = 3–5 mice. ANOVA with Tukey post‐hoc correction for multiple comparisons, ^*^ *p* < 0.05; ^**^ *p* < 0.01; ^***^ *p* <0.001; ^****^ *p* < 0.0001.

Previous studies have reported that severe trauma can have systemic effects on other compartments which can cause inflammation followed by refractory regulatory responses, possibly contributing to acute pneumonia in lungs.^[^ ^28^ ^]^ In mouse models of injury, neutrophil migration to the lung has also been reported,^[^ ^28c^ ^]^ and systemic effects such as alterations in peripheral blood leukocytes and gene expression in lymph nodes have been reported in the VML model.^[^ ^5^, ^6^ ^]^ To understand the influence of injury with and without ECM treatment on other compartments we analyzed the change in frequencies of myeloid cells from uninjured condition versus a control muscle injury and ECM_tx_ injury. In systemic circulation, we reported an initial slight reduction in eosinophil proportion at 7 dpi in both control and ECM_tx_ injury when evaluated as a fraction of total peripheral blood leukocytes albeit this was not statistically significant (*p* = 0.07, **Figure** **4**a). Unlike control injury group, the eosinophil proportion in ECM‐treated group increased significantly at 21 dpi in comparison with 7 dpi (*p* = 0.002, Figure 4a). In contrast to eosinophils, we reported a dramatic initial (7 dpi) increase in neutrophils in the blood in response to control injury from their uninjured level before declining at 21 dpi (*p* < 0.0001, Figure 4a). This was not observed in ECM‐treated group with only a modest increase at 21 dpi which was not significantly different from steady‐state uninjured mice (Figure 4a). In addition, we reported an initial (7 dpi) increase in macrophage‐like (F4/80+) and other myeloid cell (CD11b+Lin‐) frequencies in both groups from their uninjured level before returning to steady state at 21 dpi (*p* = 0.0075 for macrophage‐like cells in control injury at 7 dpi versus steady state, Figure 4a). Previous reports of neutrophil migration to the lung after injury have been reported in the case of laser‐induced liver injuries,^[^ ^28c^ ^]^ and likely pose a threat to lung health in severely injured patients. The fact that a local biomaterial may mitigate this systemic response is a novel finding that suggests that such local therapeutics may also be employed to improve outcomes in more distal tissues that may be altered by traumatic injury.

In addition to peripheral blood, there were also alterations in the immune cells within the lung after traumatic injury. Eosinophil proportions peaked early in control injuries with respect to uninjured mice though not significantly different, whereas with ECM_tx_ there was a delayed response with a peak at 21 dpi (uninjured: 4.23% ± 0.713 versus 7.828% ± 0.919 ECM_tx_, *p* = 0.0126, Figure 4b). Interestingly, when comparing to neutrophils, there was an increase early in control injuries (2.44% ± 0.414 uninjured versus 9.513 ± 0.404 control VML, *p* = 0.002) which was abrogated by local ECM treatment (4.526 ± 1.353, *p* = 0.012 versus control, *p* = 0.6 versus uninjured). Neutrophils in the lung of ECM_tx_ mice never reached levels that were seen in the control injury. As with neutrophils there was a transient increase in macrophages at 7 dpi in the lungs of mice with control injuries (2.797 ± 0.359 uninjured versus 8.785% ± 1.582 control injury, *p* < 0.0001) that was not present in ECM_tx_ mice (4.098 ± 0.611, *p* = 0.0003). Overall, there were no significant alterations in other myeloid cells (CD11b+Lin‐) when compared to uninjured mice. When evaluating the structure of lungs, overall cellularity was similar between the two treatment groups, with ECM_tx_ showing slightly higher eosin staining in granular cells suggesting the eosinophil presence as noted in flow cytometry, with control injury having fewer eosin‐containing granular cells more associated with neutrophils and other immune cells (Figure 4c).

**Figure 5:**
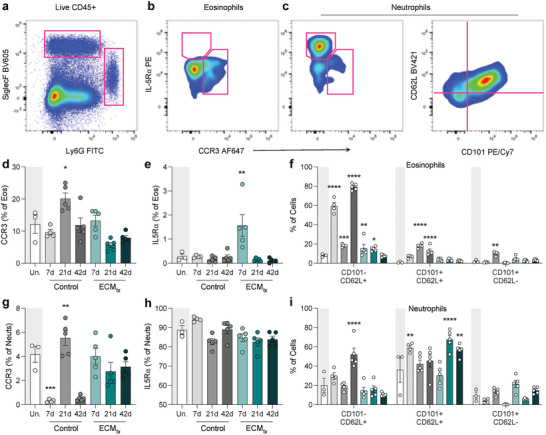
Granulocyte phenotype in lung‐responding cells after distal muscle injury. a) Example flow cytometry plot showing eosinophils and neutrophils (pink gates). b) Example gating of IL5ra+ and CCR3+ eosinophils. c) Example gating for IL5ra+ and CCR3+ neutrophils (left) and CD101+ and CD62L+ neutrophils (right). d) CCR3+ Eosinophils as a percent of total eosinophils, e) IL5ra+ Eosinophils as a percent of total eosinophils, f) CD101 and CD62L positivity as a percent of total eosinophils. g–i) CCR3, IL5ra, CD101, and CD62L positivity on neutrophils. White bar = uninjured, Black/gray bars = control injury, Teal bars = ECM_tx_. Data are mean ± SEM, *n* = 3–5 mice, ANOVA with Tukey post‐hoc correction for multiple comparisons. ^*^ *p* < 0.05; ^**^ *p* < 0.01; ^***^ *p* < 0.001; ^****^ *p* < 0.0001 compared to uninjured control.

As we evaluated granulocytes in the muscle, we evaluated the profile of cytokine/chemokine receptors and activation markers in the lung to determine if there were alterations in their phenotype in addition to their recruitment to the lung (**Figure** **5**). When evaluating eosinophils and neutrophils (Figure 5a), we saw distinct populations of IL5ra+, CCR3+, CD62L+, and CD101+ cells (Figure 5b,c) that changed after injury. Overall, CCR3 expression stayed relatively constant with some variability between mice. The only significant increase was seen at 7 dpi in control injured mice (Figure 5d). Interestingly, before peaking in proportion in the lung there was a significant increase in IL5Ra+ lung eosinophils in ECM_tx_ mice compared to an uninjured control which was not present at other timepoints or in control injury (Figure 5e). The greatest alterations in surface markers were seen on CD101‐CD62L+ eosinophils in control injury with peaks at both 7 and 42 dpi reaching as high as 80% of lung eosinophils in those mice (Figure 5f). There was also a transient and modest increase in CD101+CD62L+ macrophages in control injuries. In terms of neutrophils, there was a decrease in CCR3+ cells at both 7 and 21 dpi in control injuries that wasn't present in ECM_tx_ (Figure 5g). While we saw increases in IL5ra on eosinophils in the lungs of ECM_tx_ mice, there was no alteration in neutrophils, with the majority (>80%) of lung neutrophils being positive for IL5ra (Figure 5h). As with eosinophils, we only saw increases in CD101‐CD62L+ neutrophils in the lungs of control injury mice with a peak at 42 dpi (Figure 5g). There was an early preference for CD101+CD62L+ neutrophils in control injury mice (7 dpi peak) that was inverted with ECM_tx_ showing greater double positive fraction at 21 and 42 dpi. Systemic alterations in immune cells from distal inflammation have been reported in a number of contexts including traumatic injury in mice and humans.^[^ ^28^, ^29^ ^]^ While not for local scaffold implants, other ECM‐based biomaterials that have isolated the soluble fraction of these scaffolds have been shown to alter vascular permeability and systemic immune responses, suggesting a potential role for circulating materials in these responses in addition to cellular migration.^[^ ^30^ ^]^

### 2.5 Loss of Eosinophils Results in Changes in Cell Infiltration Along with Protein Degradation and Metabolic Pathways

**Figure 6:**
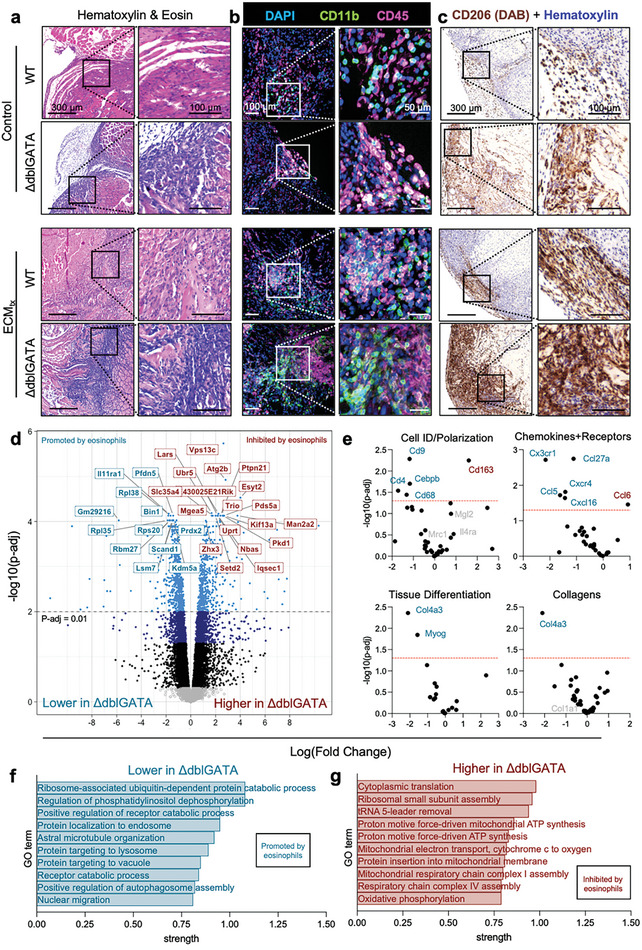
Local injury microenvironment structure is dependent upon eosinophils and altered in eosinophil‐deficient ΔdblGATA mice. a) Hematoxylin and eosin (H&E) stained sections of wild‐type (WT) and eosinophil‐deficient (ΔdblGATA) muscles at 7 days post‐injury (dpi) with and without extracellular matrix (ECM) treatment. b) Immunofluorescent staining of myeloid infiltration at 7 days post‐injury at the injury interface. Blue = DAPI/nuclei, Green = CD11b, Magenta = CD45. c) CD206 immunohistochemical staining, Brown = CD206, Purple = Hematoxylin counterstain. d) Bulk RNAseq of muscle tissue at 7 dpi of control injury displayed as fold change over WT. e) Select genes from bulk RNAseq data on immune activation and tissue differentiation. f,g) String‐db gene ontology enrichment for genes (f) decreased or (g) increased in ΔdblGATA mice compared to WT controls. Data are representative of *n* = 5 mice (microscopy) or 3 mice (RNAseq).

To evaluate the role of eosinophils on the structure of muscle tissue after injury and scaffold integration, we compared injuries in wild‐type (WT) and ΔdblGATA (eosinophil‐deficient) mice. Histologically, there appeared to be a denser cellular infiltrate at the muscle interface with the ECM materials though the amount of infiltrate into the scaffold did appear similar (**Figure** **6**a). Though eosinophils make up a significant fraction of myeloid cells responding to the injury, there was not a large decrease in CD11b+ cells at the wound space as determined by immunofluorescence microscopy (Figure 6b). Morphologically there were differences in the clustering of immune (CD45+) and specifically myeloid cells (CD11b+) with dense areas of cells as opposed to a more distributed phenotype in WT mice. Given the large proportion of immune infiltrate that are eosinophils in WT mice, if these cells did not influence the chemotaxis or phenotype of other cells in the microenvironment, we would have expected for there to be a decreased cellular infiltrate due to the loss of eosinophils, especially in terms of immune cells. This does not appear to be the case, and there is potentially a compensatory response based on the presence of CD11b+ and CD45+ cells in the scaffold microenvironment. This phenotype is possibly due to the immunoregulatory behavior of eosinophils wherein they are secreting IL‐10 to mediate a transition in the microenvironment from a pro‐inflammatory type‐1 like response to a more pro‐resolution type‐2 and regulatory response.

In order to further evaluate this phenomenon, we investigated the role of eosinophils in type‐2 macrophage polarization. Previous reports have shown that eosinophil‐secreted IL‐4 was important in cardiotoxin‐mediated muscle degeneration recovery.^[^ ^3^ ^]^ Furthermore, without Th2 T cells, macrophages are shown to have stunted M2‐like macrophage polarization as evaluated via CD206 expression.^[^ ^6a^ ^]^ To this end, we stained sections for CD206 to evaluate polarization and localization in the wound microenvironment with and without eosinophils (Figure 6c). Interestingly, there was no significant loss of CD206 expression even without the type‐2 rich IL‐4 producing eosinophils via the ΔdblGATA model. As it has been shown that CD206 is lost in *Rag^1−/−^* mice which still retain eosinophils, these data agree with previous findings that though eosinophils are the most numerous IL‐4 secreting cells in the wound microenvironment, it may be other cells, such as adaptive immune cells, that are driving some aspects of macrophage polarization, especially the M2‐like macrophage transition after initial injury. Even further, it appears that there may be some slight increase in CD206 expression especially in response to ECM treatment in ΔdblGATA mice.

Given these changes in the structure and the apparent lack of change in CD206 expression on macrophages, which is known to be dependent upon IL‐4, we further analyzed the alterations in immune response to traumatic muscle injury in eosinophil‐deficient mice through bulk RNAseq (Figure 6d–g). At 7 days post‐injury (control) there was a significant fraction of genes that were altered in ΔdblGATA mice (Figure 6d). When focusing on genes of interest such as cell polarization and cytokines/chemokines, we saw that there was minimal alteration in M2‐macrophage associated genes such as *Mrc1*, *Mgl2*, and *Il4ra*, while there was a significant increase in *Cd163* and decrease in *Cebpb* (Figure 6e). Chemokine signals were largely unchanged with a handful being decreased (*Cx3cr1*, *Ccl27a*, *Ccl5*, *Cxcr4*, *Cxcl16*) and one being increased (*Ccl6*). Tissue differentiation genes were also largely unchanged with a slight decrease in *Myog* associated with muscle regeneration and agreeing with previously published work in cardiotoxin models.^[^ ^3^ ^]^ When evaluating these changes wholistically through gene ontology enrichments, we found that there was overall a decrease in gene associated with phagocytosis and protein degradation (Figure 6f) but an increase in metabolism‐related genes in the absence of eosinophils (Figure 4g).

### 2.6 Early Myeloid Infiltration into Muscle and Lungs of Injured Mice is Largely Unaffected by the Absence of Eosinophils

**Figure 7:**
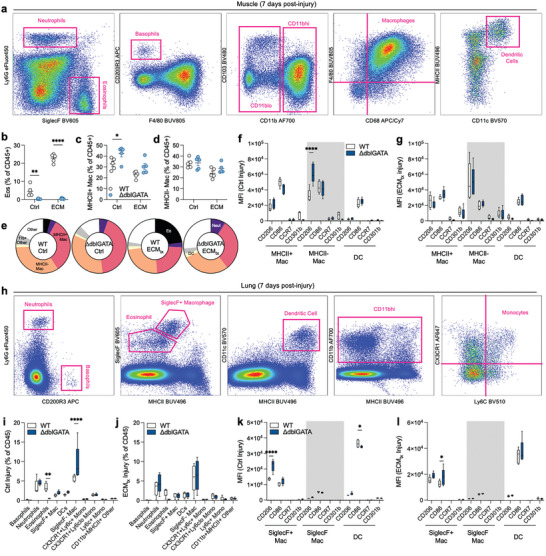
Eosinophil deficient mice exhibit typical local and systemic myeloid immune responses to traumatic injury. a) Gating scheme for myeloid cells in muscle tissue at 7 days post‐injury (dpi). b) Eosinophils as fraction of total CD45+ immune cells, c) MHCII+ macrophages as a fraction of CD45+, d) MHCII‐ macrophages as a fraction of CD45+, e) overall immune profile in control and ECM_tx_ injury in wild‐type (WT) and ΔdblGATA mice. f,g) Macrophage phenotype in f) control and g) ECM_tx_ injury, MFI = median fluorescence intensity. h) Gating scheme for myeloid cells in lung tissue at 7 dpi. i,j) Cell populations in lungs from i) control and j) ECM_tx_ injury at 7 dpi. k,l) macrophage and DC phenotype in k) control and l) ECM_tx_ injury. Data are mean ± SEM (dot plots) or interquartile range (box plots), *n* = 5. ANOVA with Tukey post‐hoc correction for multiple comparisons ^*^ *p* < 0.05, ^**^ *p* < 0.01, ^****^ *p* < 0.0001.

To further explore the immunologic response in ΔdblGATA mice, using flow cytometry we investigated myeloid infiltration in the wound space and lungs at 7 days post‐injury which is the peak of macrophage presence in the muscle wound (**Figure** **7**). Through a myeloid phenotyping antibody panel, we were able to isolate granulocytes, macrophages, and dendritic cells to characterize their response to injury (Figure 7a, gating strategy shown for WT mice). As expected, no Siglec‐F+ eosinophils were detected in the wound microenvironment of ΔdblGATA mice confirming validity of the model as an eosinophil‐deficient strain in response to muscle trauma (Figure 7b). While there was a modest increase in MHCII+ macrophages in the control injury (Figure 7c) there was no change in the more M2‐like MHCII‐ macrophages (Figure 7d) and other cell populations largely remained consistent with WT mice (Figure 7e). In terms of macrophage polarization, in control injury there was a slight increase in CD206 on MHCII‐ macrophages infiltrating control injury (Figure 7f) agreeing with our observations in histopathologic analyses. There were no significant changes in the surface markers in other cell populations or in ECM_tx_ mice (Figure 7f,g). We also evaluated the lungs with the same myeloid phenotyping panel (Figure 7h). There was a slight increase in SiglecF‐macrophages in the lung of control injured mice, but overall minimal alterations in immune cells in these mice in both control and ECM_tx_ injury (Figure 7i,j). As with local muscle tissue there was a slight increase in CD206 in control injury (SiglecF+ Macrophages, Figure 4k) and a slight increase in CD86 in ECM_tx_ injury (Figure 7l). The minimal alterations in macrophage polarization in ΔdblGATA mice when compared to previously published works on models, such as *Rag1^−/−^* which lack adaptive immune cells,^[^ ^5^, ^6^ ^]^ show that while a cell can be present in lower numbers within the injury site, they can have a large impact on cell polarization, and alternatively cells with large numbers are not necessarily major drivers of every stage of the immune response.

## 3 Conclusions

While eosinophils have been implicated in skin wound pathogenesis previously, we have shown for volumetric muscle injuries that eosinophils play a role in regulating post‐trauma immune responses but do not alter early IL‐4 dependent M2‐like macrophage polarization. Others have shown important roles for eosinophils in muscle healing, specifically after cardiotoxin injury through secretion of IL‐4. Here, we see both strong IL‐4 and IL‐10 production from eosinophils responding to a decellularized ECM‐treated muscle injury, which shows a complex phenotype of these granulocytes that could affect multiple aspects of skeletal muscle regeneration. Interestingly, the absence of eosinophils does not correlate with a decrease in *Il4* or M2‐macrophage polarization in the wound environment even with their significant presence early in response to injury. In addition to shaping the local granulocyte response, these biomaterials are able to affect distal processes such as preventing trauma‐associated neutrophil accumulation in the lungs of injured mice. Such systemic effects of local biomaterial treatment are critical to consider in complex tissue injury and multi‐organ damage that is often observed with volumetric injuries. Likely there is an importance for eosinophils during the specific stages of skeletal muscle healing, but early macrophage polarization and response is largely unaffected by their absence. Future studies are needed to evaluate downstream effects of the absence of eosinophils in the long‐term recovery from traumatic injury (VML) and comparison directly to other models such as cardiotoxin (CTX), each with their own variables such as blood vessel and lymph disruption (VML) or introduction of a foreign chemical that is less frequently seen in clinical injuries (snake venom, CTX).

## 4 Experimental Section

### ECM Preparation

Materials were generated and characterized as per previously described.^[^ ^5^, ^31^ ^]^ Briefly, small intestine was dissected out from a 5‐ to 6‐month‐old American Yorkshire pigs (Wagner Meats). The submucosa layer (SIS) was isolated by physically removing muscularis layer followed by mechanically scrapping of luminal layer. The resulting SIS was then rinsed with 10% anti‐fungal and anti‐biotic suspension (penicillin, streptomycin, and amphotericin B) in distilled water and were stored at −80 °C until decellularization. After thawing, SIS was cut into 1‐inch segment in an aseptic environment followed by 30‐min incubation in 4% ethanol (Fisher Scientific) and 0.1% peracetic acid (Sigma) with continuous stirring on a magnetic stir plate. The decellularized ECM was then neutralized with successive washes of sterile 1× PBS and distilled water. The ECM was then dried by placing on sterile absorbent pads and was then transferred to a 50 mL conical tube and frozen at −80 °C until lyophilization for 24 h. The lyophilized material was then loaded into sterile cryogenic milling containers and milled into a fine powder using a SPEX Sample Prep cryogenic milling device. The resulting powder was hydrated with sterile PBS (pH 7.2) to form a thick paste which was finally loaded into a slip‐luer 1 mL syringe for application into the trauma site.

### Volumetric Muscle Loss Surgery

Mice received volumetric muscle loss trauma in both lower limbs as per the previously described method.^[^ ^5d^ ^]^ Briefly, hairs were removed from both lower limbs of 10‐ to 12‐week‐old female C57BL/6 WT mice (Jackson Laboratory stocks: 000664) by shaving with an electric trimmer followed by application of depilatory cream, 1 day before surgery. Next day, mice were anesthetized in anesthesia chamber under 4.0% isoflurane in oxygen at a 200 cc min^−1^ flow rate and received subcutaneous injection of slow‐release buprenorphine for pain management (0.5 mg mL^−1^). The mice were then maintained at 2.0% isoflurane during the overall surgical procedure that was concluded in under 10 min. In surgical procedure, the incision site was sterilized with three rounds of betadine followed by 70% isopropanol before making a 1 cm incision in the skin and through the fascia above the quadriceps muscles. Using surgical scissors, a 3–4 mm defect was created in the mid‐belly section of the quadriceps muscle by removing 1/3 of the quadriceps muscle. After muscle removal, resulting tissue gap was filled with uniform amount (50 µL) of porcine‐derived ECM scaffold paste or with sterile 0.9% saline solution (for control group). The wound was then subsequently closed with 3–4 wound clips (7 mm, Roboz) and the procedure was repeated on the contralateral leg. After the surgery mice were placed under a heat lamp for 2–3 min to let them recover from anesthesia and surgical tools were sterilized in a glass bead sterilizer for next surgery. Mice were then placed back in the cage and monitored closely until ambulatory and grooming. They were then maintained on regular diet with enrichment until the end of the study. At predetermined endpoints, mice were euthanized by carbon dioxide asphyxiation for 5 min followed by cervical dislocation. The protocol was approved by the NIH Clinical Center Animal Care and Use Committee under animal protocol number NIBIB 20‐01.

### Flow Cytometry Staining

After the due time point, mice were ethically euthanized followed by dissection of injured muscle along with implanted scaffolds. The dissected muscle was then finely diced and digested with digestive media (0.25 mg mL^−1^ Liberase (Sigma) and 0.2 mg mL^−1^ DNase I (Roche) in HEPES supplemented DMEM) on a shaker at 100 rpm for 45 min at 37 °C.^[^ ^32^ ^]^ The digested suspension was then filtered through a 70 µm cell strainer and washed with 1× PBS and centrifuged at 350 g for 5 min at room temperature. The cell pellets were then resuspended in 1 mL 1× PBS followed by addition of 9 mL of 5 × 10^−3^ m EDTA in 1× PBS solution to reduce cell clumping. The suspension was then incubated on ice for 10 min. After, incubation, volume was adjusted to 50 mL with cold 1× PBS followed by centrifugation at 350 g for 5 min at 4 °C. Samples are resuspended in 1 mL of 1× PBS followed by 5 mL RBC lysis buffer (Buffer EL, Qiagen) to eliminate red blood cell contamination. For lungs sample, similar procedure was repeated.

Blood was collected from mice through submandibular bleed to isolate 100 µ of peripheral blood. The blood was collected in a 1.5 mL Eppendorf tube containing 250 µL of 5 × 10^−3^ m EDTA solution. After collection, the tube was then briefly vortexed to avoid clot formation. Then blood was then treated with RBC lysis buffer (EL buffer, Qiagen) as previously described^[^ ^33^ ^]^ and suspension was incubated for 15 min on ice before centrifugation and washing the cell pellet with 1× PBS.

The cell pellets were then re‐suspended in 200 µL viability dye solution (1:1000 dilution of Live/Dead Blue (Thermo Fisher) in PBS) for 20 min on ice followed by washing with wash buffer (1% BSA and 2 × 10^−3^ m EDTA in 1× PBS). The cells were treated with monocyte block (BioLegend) then stained with fluorescent antibodies (Table S1, Supporting Information, and Lokwani et al^[^ ^5b^ ^]^) followed by incubation at 4 °C for 30 min. After incubation the cells were washed three times with wash buffer and analyzed on a 5 Laser Cytek Aurora flow cytometer.

### Autofluorescence Extraction and Spectral Unmixing

Considering high autofluorescence cells such as eosinophils and macrophages in our sample, unstained sample was used to characterize three different autofluorescence population as described previously^[^ ^5^, ^34^ ^]^ (i.e., AF0, AF1, and AF2 corresponding to autofluorescence of lymphocytes, eosinophils, and macrophages, respectively). These autofluorescence signatures were used as controls along with other single stain cells or bead controls for spectral unmixing. The unmixed sample was then observed on N/N plots and minor unmixing issues were corrected by utilizing spillover correction function of Spectroflow software.

### Cell Sorting of Eosinophils

To sort eosinophils, the isolated cell suspension was stained with viability dye (7‐AAD) and flow cytometry specific antibodies (Table S2, Supporting Information) and after washing cells were diluted in 2 mL of wash buffer and loaded into the BD FACS Melody cell sorter. The CD45^+^ viable cells were further gated and CD11b^+^/MHCII^−^/Siglec‐F^+^ population was sorted in 500 µL of chilled sorting buffer (fetal bovine serum (FBS) 50% v/v in PBS). The sorted cells were then centrifuged at 400 g at 4 °C for 10 min and the cell pellet was resuspended in RLT buffer containing 1% β‐mercaptoethanol for RNA isolation. The samples that had a very low yield were directly sorted in RLT buffer to avoid loss in cell number.

### RNA Isolation and RT‐PCR

The RNA isolation of sorted eosinophils was performed using RNeasy mini column kit (Qiagen) as per manufacturer instructions after resuspension into RLT Buffer with β‐mercaptoethanol. The eluted RNA was then used for cDNA synthesis. Briefly, 11 µL of eluted RNA was added to a SuperScript IV cDNA synthesis kit reaction as per manufacturer guidelines (ThermoFisher Scientific). The resulted cDNA was then pre‐amplified by utilizing Taqman preamplification kit. Briefly, 12.5 µL of cDNA was mixed with 12.5 µL of pooled probe mixture (0.2× pool of all targeted gene primer in TE buffer) and 25 µL of Taqman preamp master mix. The resultant mixture was then subjected to thermocycling as per manufacturer guidelines. For RT‐PCR reaction, 2 µL of amplified cDNA was mixed alongside with 10 µL of Taqman Fast Advanced master mix, 7 µL of DEPC water, and 1 µL of FAM‐MGB primer/probe. The resultant mixture was then run on an Applied Biosystems QuantStudio 3 RT‐PCR Machine (ThermoFisher) for 40 cycles. The CT values of all gene probes were normalized by subtracting with GUSB CT values. ΔΔCT values were calculated by subtracting ΔCT values of each probe with average corresponding ΔCT of uninjured muscle sample.

### Cytospin Staining

Eosinophil cytospin was prepared from 100 µL of sorted cell suspension for each cytospin slide. The cytospin was stained with Epredia Shandon Kwik‐Diff Stains (Fisher Scientific) as per manufacturer guidelines. Briefly, the cytospin was fixed for 30 s in fixative solution provided in the kit followed by 30 in solution 1 (Eosin) and then 30 s solution 2 (Methylene Blue). The slide was then washed with de‐ionized water and left on bench vertically with support for drying before applying mounting media and coverslip. The stained slide was observed under light microscope (EVOS, ThermoFisher) to characterize the nucleus morphology of eosinophil and to ascertain the purity of sorted eosinophil suspension.

### Tissue Processing for Histopathology

Quadriceps muscles were dissected and placed directly into 10% neutral buffered formalin (NBF, Sigma) for 24 h prior to rinsing in distilled water and dehydrating through a graded ethanol series followed by clearing in xylenes (Sigma) and paraffin (Leica) infiltration using an automated tissue processor (Leica TP1020). Resulting samples were then bisected at the midpoint of the injury to generate a cross‐sectional face of the injury area, then mounted in a paraffin block. Five (5) micron sections were taken using a standard microtome (Leica) followed by mounting on charged slides and drying in a 60 °C oven for 2 h. Resulting slides were dewaxed and rehydrated in graded ethanol series then either stained with methods below for immunohistochemistry or with H&E as per manufacturer's instructions (Sigma). H&E slides were then imaged in brightfield using a ThermoFisher EVOS microscope.

### Immunofluorescent and Immunohistochemical Staining

Rehydrated slides were subjected to citrate‐based antigen retrieval (Sigma) for 20 min in a vegetable steamer followed by slow cooling for 20 min on a benchtop. Samples were washed in 0.05% Tween 20 in 1× Tris buffered saline (TBS) followed by three rinses in 1× PBS and outlined with a hydrophobic barrier pen. For CD206 IHC staining, slides were incubated in 3% hydrogen peroxide for 10 min at room temperature prior to blocking. All slides were blocked in 1% BSA (Fisher) + 4% normal goat serum (NGS, Cell Signaling Technologies) + 0.05% Tween 20 (Sigma) in 1× PBS for 1 h at room temperature prior to incubating with primary antibody (CD206 clone E6T5J, Cell Signaling Technologies, 1:200 dilution; CD45 clone S18009F, BioLegend, 1:100 dilution; CD11b clone EPR 1344, Abcam, 1:1000 dilution) diluted in blocking buffer overnight in a humidity tray at 4 °C. The following morning samples were washed three times in 1× PBS + 0.05% Tween20. For CD206 IHC staining, samples were stained using the Vector ABC Kit as per manufacturer's instructions, exposed to DAB substrate (Vector DAB EqV) for 1 min and 20 s, followed by counterstaining in Harris hematoxylin (Sigma) for 5 min with a destain in acid alcohol followed by dehydration and mounting in a synthetic resin (Permount, Fisher Scientific). For CD45 and CD11b immunofluorescent stains, slides were incubated in 1:250 secondary antibody (anti‐Rat AF488, anti‐Rabbit AF647, ThermoFisher) for 2 h at room temperature in the dark prior to washing three times in 1× PBS. Samples were then stained with DAPI for 5 min prior to autofluorescence blocking using Vector TrueView Autofluorescence Quenching Kit (as per manufacturer's instructions) and mounting in the accompanying mounting medium. Slides were imaged on a Nikon ECLIPSE Ti2‐E wide‐field inverted fluorescent microscope.

### Bulk RNA Sequencing

Bulk RNA sequencing analysis was performed as described previously.^[^ ^5c^ ^]^ Mouse quadriceps were dissected and flash frozen at 7 dpi. RNA sequencing was performed at Azenta Life Sciences. Data analysis was performed on paired end reads from three independent samples per group. For each sample, Basecall accuracy was verified to be >30 and pseudoalignment was performed using Kallisto.^[^ ^35^ ^]^ Transcript abundance files were then imported into R (R4.4.1) and assigned gene identity using the package Ensmbldb. Counts per million (CPM) values were determined using EdgeR and filtered using CPM > 1 in at least three samples. Counts were then log transformed and normalized using the Trimmed Mean of M‐values method. Differential expression analysis was carried out using the package limma.

### Statistical Analysis

Animals were randomly assigned to treatment groups prior to performing procedures and sample size was determined based on prior experience with the animal model. For comparison of population means, an alpha value of 0.05 was used. Statistical analysis for flow cytometry and qRT‐PCR data was performed using two‐way ANOVA with Tukey's post hoc correction for multiple comparisons. Please see bulk RNA sequencing section for methods utilized for RNA sequencing data analysis. Please see figure legends for *p*‐value signifiers.

## Conflict of Interest

The authors declare no conflict of interest.

## Author Contributions

R.L., D.F., D.R.H., T.B.N., and A.J. performed experiments. R.L. and K.S. analyzed data and wrote the manuscript. All authors reviewed and edited the manuscript. K.S. provided funding and oversaw the study.

## Supporting information

Supporting Information

## Data Availability

The data that support the findings of this study are available from the corresponding author upon reasonable request. RNAseq data are available through GEO, GSE272081.
