# Supplementary material for: Eosinophils Respond to Extracellular Matrix Treated Muscle Injuries but are Not Required for Macrophage Polarization
Source: Adv Healthc Mater. 2024 Jul 27;14(5):2400134. doi: 10.1002/adhm.202400134 (PMC11834370; doi:10.1002/adhm.202400134)
Supplement: Supplementary file 1 — Supporting Information [file ADHM-14-0-s001.docx]

Supporting Information

**Eosinophils respond to extracellular matrix treated muscle injuries but are not required for macrophage polarization**

*Ravi Lokwani, Daphna Fertil, Devon Hartigan, Aditya Josyula, Tran B. Ngo, Kaitlyn Sadtler*

| **Antibody** | **Dilution** |
| --- | --- |
| Live/Dead Blue | 1/1000 |
| CD45-BUV805 | 1/100 |
| CD101-PE-Cy7 | 1/400 |
| CCR3-AF647 | 1/100 |
| Siglec F -BV650 | 1/200 |
| Ly6G- FITC | 1/200 |
| CD125-PE | 1/400 |
| CD62L-BV421 | 1/100 |
| F4/80-BUV563 | 1/100 |
| CD11b-AF700 | 1/400 |

**Supplementary Table 1: Antibodies used in flow cytometry analysis.**

| **Antibody** | **Dilution** |
| --- | --- |
| 7AAD | 1/1000 |
| CD45-AF488 | 1/100 |
| CD11c-PE | 1/100 |
| CD11b-BV510 | 1/100 |
| MHCII-BV421 | 1/100 |
| F4/80 APC-Cy7 | 1/100 |
| Siglec F BV786 | 1/100 |

**Supplementary Table 2: Antibodies used in flow sorting of eosinophils.** Eosinophils were identified as 7AAD-CD45+CD11c-CD11b+MHCII-F4/80-SiglecF+.
